# Optimizing a Patient-Centered Report of Somatic and Germline Genetic Test Results

**DOI:** 10.1200/PO-24-00927

**Published:** 2025-04-18

**Authors:** Akila Anandarajah, Hank Dart, Bailey Martin-Giacalone, Melinda Bacchini, Bettina F. Drake, Graham A. Colditz, Ryan C. Fields, Li Ding, Erin Linnenbringer

**Affiliations:** ^1^ Division of Public Health Sciences, Department of Surgery, Washington University School of Medicine, St Louis, MO; ^2^ The Cholangiocarcinoma Foundation, Riverton, UT; ^3^ Department of Surgery, Washington University School of Medicine, St Louis, MO; ^4^ Department of Medicine, Washington University in St Louis, St Louis, MO; ^5^ McDonnell Genome Institute, Washington University in St Louis, St Louis, MO; ^6^ Division of Oncology, Washington University in St Louis, St Louis, MO; ^7^ Department of Genetics, Washington University in St Louis, St Louis, MO; ^8^ Siteman Cancer Center, Washington University in St Louis, St Louis, MO

## Background

Although there is a growing emphasis on returning research results to participants,^1^ genetic findings are often difficult for patients and nonspecialists to understand. Communication challenges are even greater in precision oncology as patients' test results may include somatic variants that are found only in the patient's tumor and/or germline variants that could be shared by other family members.^2,3^ Previous work has developed reports that are user-friendly; however, these efforts are largely confined to gene panels for a single disease and do not include somatic findings.^4^ An objective of the Washington University Participant Engagement and Cancer Genome Sequencing (WU-PE-CGS) study is to fill this gap by building a participant-centered result report that summarizes somatic and germline genetic test results.

**FIG 1.**
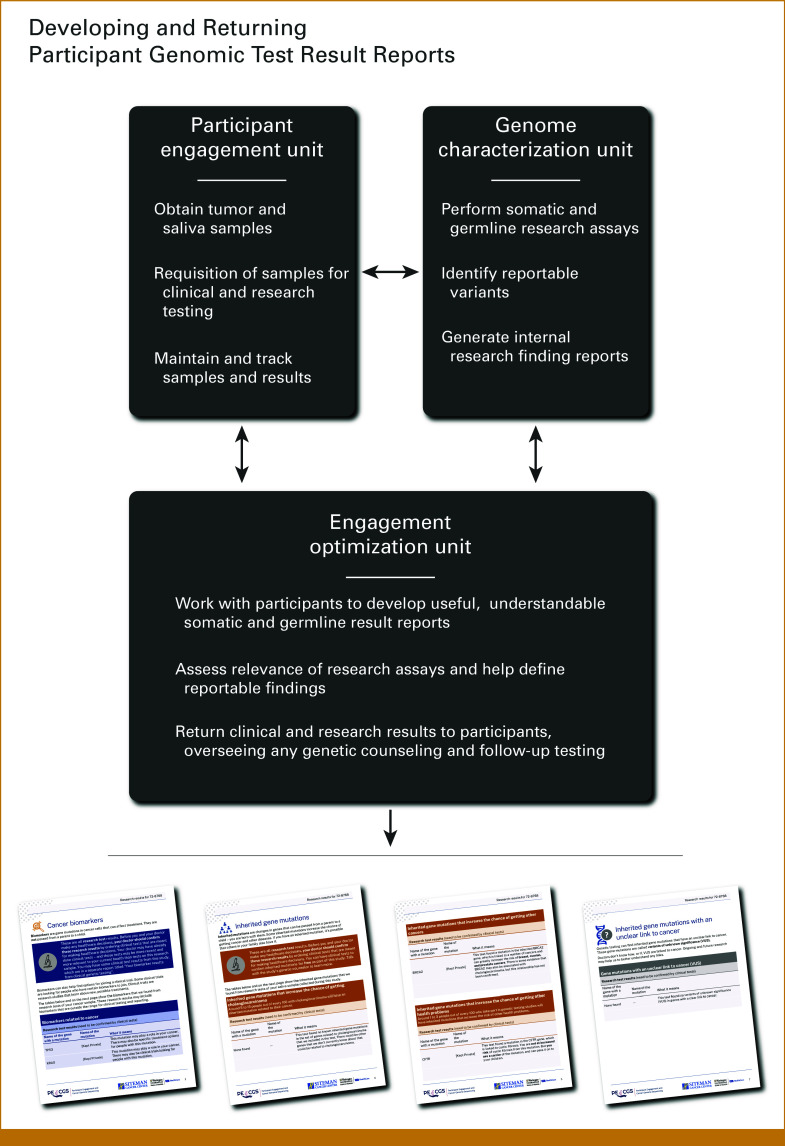
Units of the WU-PE-CGS and their role in obtaining and reporting research results to participants. WU-PE-CGS, Washington University Participant Engagement and Cancer Genome Sequencing.

The WU-PE-CGS is one of five research centers funded by the National Cancer Institute in response to the goals identified in the Cancer Moonshot^5^ and comprises three units: the Participant Engagement Unit (PEU), Engagement Optimization Unit (EOU), and Genome Characterization Unit (GCU; Fig 1). Each unit has different aims to achieve the study's goals of engaging participants directly in cancer genetic research and advancing the genetic characterization of rare, understudied cancers, particularly those affecting under-represented populations. Given that all genetic sequencing of participant samples in the WU-PE-CGS is performed in house and the GCU had not previously developed patient-facing reports, we had the opportunity for innovation.

## Report Design Principles

While designing the reports, we leveraged user-centered design principles, which can enhance communication and subjective understanding of complex information.^6^ User-centered design prioritizes the needs, preferences, and experiences of end users by actively involving them in the design and development process to ensure that the final product or solution meets their requirements and expectations.^7^ Iterative collaboration, feedback collection, and communication tailored to specific user groups are core principles of user-centered design.

Previous studies using user-centered design have demonstrated the importance of including patient perspectives when designing materials to convey complex genetic information,^6,8^ which can improve patients' experience with receiving genetic test results.^9^

We used a collaborative and iterative report development process. We engaged in extensive and ongoing collaborations with health literacy experts, study team members of the three research units, and the Participant Engagement Advisory Board (PEAB), a board of WU-PE-CGS advisors comprising study participants and patient advocates. Collaborating with diverse parties helped provide a comprehensive and multidisciplinary perspective on communicating genetic test results. The PEAB provided insights and feedback from the participant's point of view, leading to revisions that more effectively meet their needs. For example, the PEAB helped develop a plain language explanation of variants of unknown significance (VUSs) and recommended clarifying the implications of having no identified somatic or germline variants. The GCU contributed their expertise in variant interpretation and reporting. The PEU and EOU offered guidance on enhancing participant experience and engagement. Health Literacy Media, a nonprofit organization specializing in improving health literacy and creating patient-centered materials, enhanced the readability of the reports through the use of plain language and design best practices. With their input, we were able to report complex genetic information at a ninth-grade reading level.

To enhance the clarity of our reports, we aligned our language with recommendations of the Consistent Testing Terminology Working Group—a group dedicated to advocating for the uniform use of common and clear terms in biomarker (somatic) and inherited (germline) genetic testing. Their analysis of patient-identified barriers to receiving testing that revealed how inconsistent use of terminology for genetic testing results hinders effective patient communication with health care providers.^10^ For example, biomarker testing has also been referred to as molecular testing, tumor profiling, or genomic testing of cancer cells.^11(p1564)^ This inconsistent terminology is a source of confusion that impedes patients' communication with providers and comprehension of testing implications for their care. As a result, the working group recommended the following terms: “biomarker testing” for tests identifying characteristics, targetable findings, or other results from malignant tissue or blood and “genetic testing for an inherited mutation” and “inherited cancer risk” for tests identifying germline mutations.^11^ We followed these recommendations throughout our report.

To further enhance clarity, we incorporated specific design elements. Each section of the report was visually distinguished with distinct colors: biomarker results pages featured a blue color scheme for callout boxes and tables, inherited mutations pages used an orange color scheme, and VUSs used a gray color scheme (see the sample report available in the Data Supplement). Icons are also used to signal distinct areas of the report. Feedback from the PEAB motivated the consolidation of all relevant biomarkers into a single table.

## Report Structure/Content

### Introduction

The title page provides an overview of the purpose of genetic testing and describes the types of results that may appear in the report. It explains the difference between research and clinical testing and emphasizes that research results must be confirmed via clinical laboratory testing before they can be used to make health care decisions. While the report is designed so it can be viewed independently by participants, individuals with inherited mutations or VUS review the report with a genetic counselor. All other participants receive the report from a study coordinator but can request a free genetic counseling appointment.

### Biomarker Results

The next section of the report contains a definition of biomarkers and a description of how biomarkers can be useful in clinical decision making. Biomarker results are presented in a single table containing both biomarkers pertaining to the individual's cancer type and pan-cancer biomarkers. The use of a single table for these results was based on feedback from the PEAB. The column titles in the table are as follows: “name of the gene with a mutation,” “name of the mutation,” and “what it means.” The “what it means” column lists a lay language interpretation of the variant priority category classification to provide context for the participant.

### Next Steps

This section summarizes next steps for participants based on biomarker results. These include talking with the participant's cancer care team about their results, confirming research results with clinical testing before making health care decisions, and calling health insurance companies for questions regarding coverage for confirmatory testing.

To develop the recommended next steps, we incorporated various factors, including clinician expertise, study objectives, practice guidelines, and feedback from the PEAB. The recommendations from the PEAB and health professionals were essential for tailoring the next steps to best offer participants practical guidance. We aimed to ensure that participants would be provided with clearly defined actions based on their type of research results. With the study enrolling individuals at various treatment stages, participants might have already undergone clinical testing before enrolling in the study. We accounted for this when structuring the next steps to ensure that participants receive appropriate guidance and support.

### Inherited Mutation Results

The next section in the return of results report includes a plain language explanation of inherited mutation results. Results are reported in three tables: (1) variants associated with the participant's cancer type, (2) variants in pan-cancer genes, and (3) variants related to other medical conditions, as recommended by the American College of Medical Genetics and Genomics.^12^ Participants may opt out of learning about any or all inherited findings, and the report is adjusted according to their preferences. Recommended next steps related to inherited mutation findings include speaking with a WU-PE-CGS genetic counselor and sharing the results with participants' cancer care team and family members. Specifically, individuals identified as having an inherited mutation receive information about additional resources available for family members, options for further clinical confirmation through the WU-PE-CGS study, and other relevant information. A different next step page is provided to participants who have no identified inherited mutations. All participants receive information about the role of a genetic counselor and how to contact the WU-PE-CGS genetic counselor free of charge.

### VUS Results

The next section defines VUS and reports any inherited VUS found in the research genetic testing. Initially, the WU-PE-CGS return of results team questioned returning VUS findings to participants because of the uncertainty surrounding their clinical significance. However, PEAB members strongly advocated for the inclusion of VUS findings, citing the importance of transparency and providing participants with a complete picture of their genetic data. In response to this feedback, the research team decided to return VUS findings in established cancer genes; return of VUS findings in preliminary cancer genes is based on additional review by a study genetic counselor. The team worked diligently to develop a robust and patient-friendly explanation of VUS findings in collaboration with the PEAB.

Recommended next steps based on VUS findings included talking with the participant's cancer care team about their results and confirming results with clinical testing meant for health care decisions. Participants are given information about the role of a genetic counselor and how to contact the WU-PE-CGS genetic counselor if they have additional questions.

### Further Information

The final section provides information about how the study performed genetic tests and which genes were tested for inherited mutations; the name, e-mail, phone number of the study genetic counselor; a short glossary of key terms; and a resource list to learn more about genetic testing, clinical trials, WU-PE-CGS, and the participant's specific cancer type.

## Challenges and Future Directions

The WU-PE-CGS report returns genetic research findings from testing that is not conducted within a College of American Pathologists (CAP)/Clinical Laboratory Improvement Amendments (CLIA) environment. This is an important distinction as research laboratories like the GCU are not held to the CAP accreditation and CLIA regulatory requirements for generating reports and including specific data elements.^13^ With this flexibility, we were able to refine the design and content of our research finding report to meet the needs of our participants.

However, the WU-PE-CGS also offers participants results for a select list of currently clinically actionable biomarkers that are generated separately in a CAP/CLIA-certified laboratory using their standard reporting procedures. While providing potentially valuable clinical data to participants, these reports could not be modified to incorporate user-friendly design elements because of the nature of the CAP/CLIA informatics pipeline and limitations of clinical laboratory information management systems reporting modules. To provide continuity with the research report and further context for the CAP/CLIA report content, we created a cover page providing context for the clinical results in the style of the research result report.

Another challenge is building a report generation system that can handle a large volume of data efficiently and reliably. To reach a wider population, the system needs to be designed for scalability and capable of handling larger data sets without sacrificing accuracy or speed.^14^

Integrating data from multiple sources, such as different laboratories and testing pipelines, is also crucial. Ensuring that the system can handle and streamline the integration of diverse data inputs is a critical aspect of generating a comprehensive and accurate patient-centered report. This requires robust data management strategies and effective communication between different data sources.^15^ Considering the future of this work and patient-centered reporting, ongoing efforts should focus on further optimizing data integration, scalability, and regulatory compliance.

In conclusion, there is a clear need for patient-centered genetic testing reports that prioritize its users, easily facilitating an individual's comprehension of their own research and clinical results.

Genetic testing reports should use intuitive design elements and a range of information and resources to provide participants with a comprehensive and accessible overview of their genetic findings, allowing them to make informed decisions about their health care and take appropriate actions based on their circumstances.

Through collaboration and refinement, the WU-PE-CGS has developed patient-centered reports that provide clear results. We will continue to solicit feedback from patients, health care providers, and other stakeholders to identify areas of the report that require further modification and adjust the report accordingly. Only by doing so can we elevate the participant experience, enabling them to make informed decisions about their health and advancing participant engagement in genetic testing.
